# An integrative process model of resilience in an academic context: Resilience resources, coping strategies, and positive adaptation

**DOI:** 10.1371/journal.pone.0246000

**Published:** 2021-02-02

**Authors:** Dayna J. Fullerton, Lisa M. Zhang, Sabina Kleitman

**Affiliations:** School of Psychology, University of Sydney, NSW, Australia; Aalborg University, DENMARK

## Abstract

Tertiary study presents students with a number of pressures and challenges. Thus, mental resilience plays a key role in students’ well-being and performance. Resilience research has moved away from conceptualising resilience as a *trait* and towards studying resilience as a *process* by which resources protect against the negative impact of stressors to produce positive outcomes. However, there is a lack of research in the academic domain examining the mechanisms underlying this process. This study addressed this gap by examining a range of personal resilience resources and their interaction with coping responses to produce positive adaptation outcomes, in a sample of 306 undergraduate students. Firstly, individual differences in resilience were examined, whereby factor analysis resulted in self-report measures of resilience-related attributes converging onto an overarching factor. The extracted factor was then validated against markers of positive adaptation (mental well-being, university adjustment, and somatic health symptoms), and the mediating roles of coping strategies were investigated through structural equation modelling. The resilience resources factor directly predicted mental well-being and adjustment; and indirectly predicted adjustment and somatic health symptoms through support-seeking and avoidant coping, respectively. These findings have theoretical implications for how resilience is conceptualised, as well as practical implications for improving student well-being and adjustment through promoting social support and reducing disengaged and avoidant coping strategies.

## Introduction

Hardship, stress, and adversity are an inevitable part of the human experience. The study of mental resilience aims to elucidate why certain individuals are better able to withstand these experiences. One such experience which presents several challenges is tertiary-level study. University students face competing academic, social, and financial pressures and setbacks. Hence, resilience plays a fundamental role in student well-being and success [1]. The present study uses this apt context to test a novel, comprehensive model of resilience which integrates and extends upon existing frameworks.

Resilience is a broad concept lacking a universally accepted definition [2]. However, two core concepts have been commonly implicated: adversity and positive adaptation [3]. For example, the American Psychological Association [4] define resilience as “the process and outcome of successfully adapting to difficult or challenging life experiences, especially through mental, emotional, and behavioural flexibility and adjustment to external and internal demands”. Zautra and Reich [5] note three adaptive outcomes which characterise resilience: recovery, sustainability, and growth. As Fletcher and Sarkar highlight, what constitutes positive adaptation, depends on the nature of the adversity; which varies from major traumatic events to common everyday stressors [3]. Fletcher and Sarkar integrate common themes in the literature to broadly define resilience as “the role of mental processes and behaviour in promoting personal assets and protecting an individual from the potential negative effects of stressors” [3, p. 16].

Further debate surrounds whether resilience should be conceptualised as a stable trait or a dynamic process. Early research focused on resilience as a set of characteristics which buffer against the negative effects of stress [e.g., 6, 7]. Accordingly, resilience has typically been measured by self-report scales capturing the features of the way a person *is* which contribute to their resilience [e.g., 7, 8]. However, conceptualising resilience as a *trait* limits our understanding of how people respond in different situations. The view that resilience is a *process* by which personal resources (or protective factors) interact in the context of some adversity has become increasingly favoured. Still, much of the literature has focused on identifying personal resilience resources. There is a critical need to examine the role of these resources in the overall resilience process [9]. There has been considerable conceptual work undertaken to advance our understanding of resilience. However, the lack of empirical work collectively examining resilience resources in the context of the overall process presents a major gap in the field.

### General theoretical frameworks

Following the paradigm shift from resilience as a stable trait to a dynamic process, several conceptual models have been developed to describe the latter. There are a number of aspects upon which these different theories converge. The need for a generic model outlining the mechanisms which can be applied and validated in different contexts (e.g., education, sport, defence, organisational) has been noted as a critical area for advancing resilience research [3].

A key theory guiding resilience research is Richardson’s metatheory of resilience [10]. This theory postulates that resilience resources determine whether stressors cause disruption to one’s biopsychospiritual balance. If there are insufficient resources, disruption occurs, facilitating emotional and behavioural responses. These responses then lead to one of four outcomes: (1) resilient reintegration, where the individual returns to a higher level of homeostasis, (2) homeostatic reintegration, where the individual returns to their baseline level, (3) reintegration with loss, leading to a lower level of functioning, and (4) dysfunctional reintegration, leading to maladaptive and destructive behaviours.

Mancini and Bonanno’s individual differences model [11] draws on similar concepts. Whilst developed in the context of interpersonal loss, it has been adapted to other contexts such as work, sport, and everyday stress. They posit that factors involved in the resilience process converge onto common mechanisms including individual differences, appraisal processes, use of social resources, and coping strategies. They propose that individual differences such as personality and beliefs influence one’s response (i.e., selection of coping strategies) both directly, and indirectly, through appraisal processes and social resources. Effective responses then lead to minimal symptoms and positive adaptation. In comparison with other theories, Mancini and Bonanno conceptualise and operationalise resilience as an *outcome* following a stressful event. However, Dunkel-Schetter and Dolbier [12] provide several arguments for precluding this approach in favour of a process view. First, the outcomes that researchers focus on may vary considerably, leading to a lack of definitional consistency. Second, this view ignores the impact of situational factors. Finally, a process view permits greater examination of predictors and prevention than is possible when inferring resilience based on an end point. Nevertheless, this model postulates the general shared mechanisms involved in the overall process, allowing researchers to adapt the model to their context of interest by identifying specific relevant constructs, as suggested by Dunkel-Schetter and Dolbier [12].

Fletcher and Sarkar’s grounded resilience theory in the sporting context similarly postulates that protective factors interact with metacognitions and appraisals to promote facilitative responses to a stressor [13]. Leipold and Greve also propose an integrative model of coping and resilience whereby personal and situational factors influence coping processes [14]. Whilst the resilience literature at first appears to be inundated with varying theories, a closer examination reveals that there are indeed similarities between seemingly different frameworks. At the most general level, each of the discussed theories outline the influence of personal attributes and protective resources on the way one responds to a stressor, producing a particular outcome.

### Academic resilience

Academic [sometimes *educational]* resilience has been defined as an increased likelihood of educational success despite adversity [15, 16]. Martin and Marsh [16] found five factors predicted scores on a self-report academic resilience scale in a series of path analyses, which informed their 5-C model of academic resilience: confidence (self-efficacy), coordination (planning), control, composure (low anxiety), and commitment (persistence). These findings provide a starting point for identifying resources involved; however, they are confined by the view of resilience as a personal capacity. Further examination is necessary to advance our understanding of how these resources interact in the overall resilience process. In subsequent studies, Martin and Marsh introduced the concept of academic buoyancy, defined as “student’s ability to successfully deal with academic setbacks and challenges that are typical of the ordinary course of school life” [17 p. 54]. They argue that academic buoyancy and resilience are distinct constructs, where resilience relates to more extreme adversity, whilst buoyancy reflects everyday challenges. How the resilience construct relates to stressors of all degrees remains a point of empirical investigation.

Another model proposed in the academic domain is Dunn et al.’s conceptual model of medical students’ well-being [18]. They postulate that positive (e.g., support, healthy activities, mentorship) and negative (e.g., stress, time and energy demands) inputs either replenish or drain one’s ‘coping reservoir’, consisting of personality traits, temperament, and coping style. This then leads to either increased resilience or burnout. This model better captures the dynamic process through which resources interact to produce outcomes, and provides pathways through which resilience may be improved and developed. However, it has not been substantiated with empirical support.

Much of the empirical work in the education field has focused on resilience as a capacity or ability. To the best of our knowledge there appears to be no empirically supported *process* model of resilience in this domain. However, established general frameworks can be adapted and applied in different contexts. This approach, indeed, may be more beneficial for the field, allowing for greater consistency and comparisons between different contexts. Providing the empirical basis for an integrative model of resilience is the focus of this research.

### Measuring resilience

Inconsistencies in the way resilience has been conceptualised have hindered a unified approach to its measurement. The construct has been operationalised through varying ways, including (1) self-report scales, (2) indirect inferences based on core components (risk/adversity and positive adaptation), and (3) measuring adjustment to everyday stressors or experimentally-created stressors [19]. Whilst the literature has moved towards a process conceptualisation, standardised scales take a trait-like approach, focusing on characteristics within the individual [20]. Several scales have been developed and validated, with the Connor-Davidson Resilience Scale (CD-RISC) receiving the most attention [see 21, 22 for reviews]. Connor and Davidson drew upon work on the characteristics of resilient people to develop the CD-RISC [8]. Their data revealed a 25-item five-factor model. However, subsequent studies were unable to replicate these factors [23, 24]. Campbell-Sills and Stein [24] established a 10-item version; which has demonstrated stronger psychometric properties and a consistent unidimensional structure [20, 23].

Several studies in the academic domain have utilised both versions of the CD-RISC as well as other self-report measures of general resilience, including Wagnild and Young’s (1993) Resilience Scale [7] and Friborg et al.’s Resilience Scale for Adults [25]. There have also been attempts to develop academic-specific resilience scales [15, 17]. Much resilience research, particularly in the academic domain, have used measures capturing only one component of the resilience process; that is, personal resources. Other academic studies have inferred resilience based on an outcome, such as academic achievement [26]. Hence, resilience research in the academic domain needs to advance toward assessing each of the proposed components of the process.

### The present research

The current study sought to examine the mechanisms involved in the resilience process through integrating theoretical frameworks and adapting them to the academic context. To examine these mechanisms, a theoretically- and empirically-guided range of measures assessing resilience-related resources, coping responses, and academic and non-academic outcomes were selected. We then tested a path model, strongly guided by theory, investigating the relationships between the focal variables of interest. A comprehensive selection of other known predictors of coping behaviour and well-being outcomes were controlled for to assess the unique role of the resilience facets of interest whilst accounting for other common factors.

#### Resilience resources

*Mental toughness*. This construct has mostly been applied to the sport domain; though, it is receiving increasing attention in other achievement contexts such as education [27]. It is conceptualised as a personal capacity to produce consistently high levels of performance despite challenges and adversities [27]. This distinguishes mental toughness from resilience, which involves factors beyond the self and their interaction. However, given their similarities in dealing with challenges, they have been used interchangeably. As such, mental toughness may act as a protective factor in the resilience process. Indeed, Gerber et al. found mental toughness moderated the relationship between stress and depressive symptoms in high school and undergraduate students [28].

*Self-esteem*. Self-esteem refers to how one perceives and evaluates their self-worth. It has been considered a protective resource which buffers against negative impacts of challenging experiences [12]. This is supported by a large body of empirical research showing self-esteem is associated with greater resilience and overall happiness [e.g., 1, 29].

*Self-efficacy*. Self-efficacy concerns how one perceives their capabilities [30]. Martin and colleagues’ studies found self-efficacy to be a significant predictor of academic resilience in high school students [16, 31]. Similarly, Cassidy [32] found academic self-efficacy predicted academic resilience in tertiary students. Self-efficacy is thought to be dependent on the situation. Accordingly, we assessed academic self-efficacy in this study.

*Optimism*. Dispositional optimism refers to the tendency to hold positive expectations about the future. Optimists are proposed to respond proactively to adversity, and demonstrate greater persistence towards goals in challenging times [33]. Research has demonstrated an association between optimism and better mental and physical health in the face of adversity [34], including in college students [35].

*Meaning in life*. Meaning in life is defined as making sense of, or seeing significance in one’s life, as well as perceiving oneself to have a purpose or overarching aim in life [36]. Studies have supported the idea that meaning in life is protective in the face of adversity by demonstrating links with lower depression, anxiety, and stress [37, 38], and greater life satisfaction [39]. Meaning in life has been identified in resilience models in the context of loss [7, 12] though is yet to be considered in the academic context.

*Adaptability*. Martin et al. [40] define adaptability as the capacity to adjust thoughts, behaviours, and emotions in response to changing or uncertain circumstances. In a qualitative study of high achieving athletes, adaptability was identified as a key quality which enabled them to overcome challenges by solving problems creatively, learning novel work practices, and adapting positively to change [41]. Transitioning to university gives rise to a number of situations where the ability to adapt is critical, as students navigate a new environment, higher academic demands, different learning and teaching styles, as well as changing living, work, and financial situations [42]. Hence, we theorise that adaptability enhances resilience in students.

#### Appraisals and responses to stressors: Coping strategies

Resilience process theories have posited that personal attributes interact with the way one appraises and responds to stressors to influence the outcome [9]. These cognitive and behavioural efforts to manage different demands are referred to as *coping*. Hence, we employed measures of coping styles to investigate these relationships. Previous research has shown links between resilience and problem-focused coping strategies, which involve practical attempts to reduce or eliminate the stressor [43, 44]. Specifically, research suggests resilience is associated with greater use of planning [16], support seeking [45], and less use of maladaptive and avoidant strategies [43]. Whilst such relationships have been established, the mediating function of coping styles on the relationship between resilience resources and outcomes has received less attention. The present study addresses this gap by examining a range of coping strategies and their roles in the resilience process in the academic context.

#### Positive adaptation (outcomes) at university

Contextually relevant indices of positive adaptation must be considered to contextualise the resilience process. In the present study, we assessed three outcome measures. Firstly, university adjustment, which assesses how one is functioning academically, socially, and psychologically at university [46]. Secondly, mental well-being, which capture positive aspects of mental health in a general sense [47]. Lastly, we assessed somatic health symptoms to capture physical well-being.

#### Aims and hypotheses

**Fig 1:**
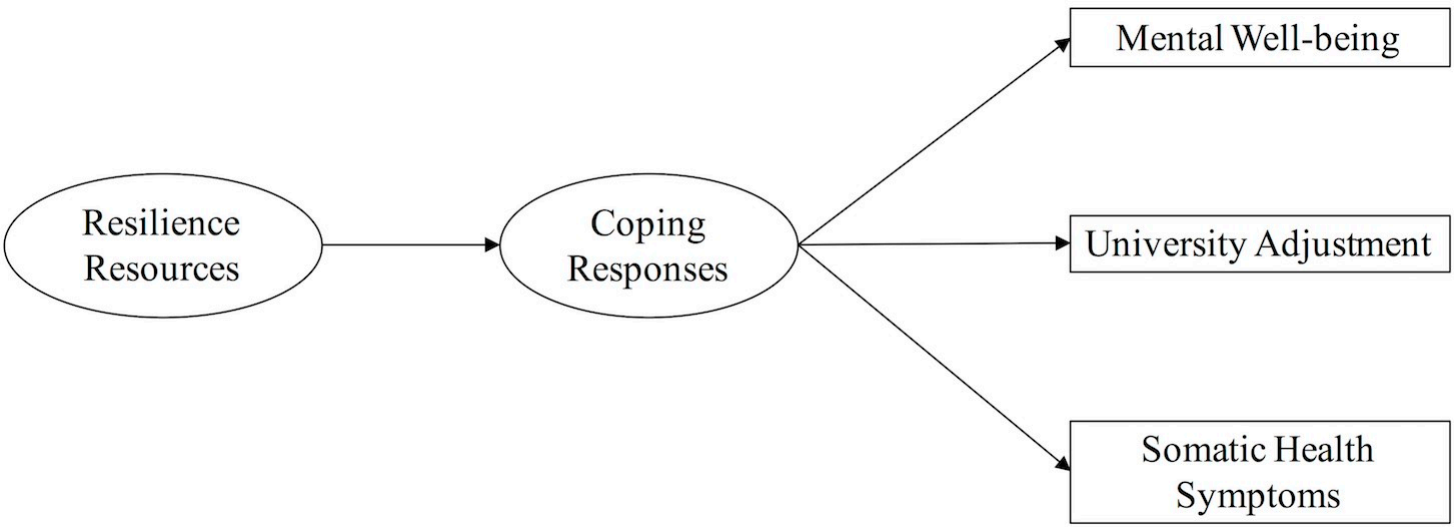
Path model to be tested.

Exploratory Factor Analysis (EFA) was used to examine the convergence of resilience-related measures assessing personal attributes and resources. Another EFA determined the convergence of coping strategies. The resulting factor(s) were included in a path model with coping responses as mediators, and positive adaptation measures as outcomes (see Fig 1).

The three aims and hypotheses were:

1. To examine the convergence of a theoretically- and empirically-guided selection of self-report measures of resilience and related constructs capturing personal resilience resources
  - Hypothesis 1: All measures will converge onto an overarching factor (i.e., resilience resources).
2. To examine the predictive validity of the broad resilience resources factor(s) on markers of positive adaptation (outcome variables).
  - Hypothesis 2.1: Resilience resources will predict greater mental well-being.
  - Hypothesis 2.2: Resilience resources will predict higher university adjustment.
  - Hypothesis 2.3. Resilience resources will predict less somatic health symptoms.
3. To examine whether coping responses mediate the relationship between resilience resources and outcome variables.
  - Hypothesis 3: Coping factor(s) will mediate the relationships between resilience resources and outcome variables.

For each hypothesis, covariates and known predictors were controlled for. These included age, gender, Big 5 personality [48], intelligence [49], and financial and living factors [50].

#### Statistical analyses

Pearson correlations and Exploratory Factor Analysis (EFA) were used to examine the factorial structure of resilience resource measures. An EFA was also performed on the coping measures to determine the underlying dimensional structure for their use in the path model. Path analysis was then performed using the extracted factors, outcome measures, and control measures to test the hypothesised model (see Fig 1).

## Method

### Participants

306 Australian psychology undergraduate students (76.1% female; mean age *=* 20.05, *SD* = 3.09) participated in return for course credit. Ethics approval was granted by The University of Sydney Human Research Ethics Committee (protocol 2019/315).

### Measures

#### Self-report resilience and related resources

*Connor-Davidson resilience scale short version* [24]. This 10-item unidimensional scale measures self-perceived ability to adapt to adversity. Items such as “I can deal with whatever comes” were rated on a 5-point scale from 0 (*not true at all)* to 4 (*nearly always true)*. The scale has previously demonstrated good reliability with an internal consistency estimate of .85 [24].

*The resilience scale* [7]. This 25-item scale assesses two dimensions of individual resilience: (1) Personal Competence, defined by self-reliance, independence, and perseverance, and (2) Acceptance of Self and Life, representing adaptability, and a balanced perspective of life despite adversity. Participants rated items such as “I usually take things with stride” on a scale from 1 (*disagree)* to 7 (*agree*). The scale has demonstrated good reliability with internal consistency estimates from .76 to .91 [7].

*Mental toughness index* [27]. This 8-item scale measures the capacity to perform well despite challenges. Participants rated items such as “I strive for continued success” from 1 (*false 100% of the time)* to 7 *(true 100% of the time)*. The measure has demonstrated good reliability with estimates of .86 to .89 across multiple samples [27].

*Rosenberg self-esteem scale* [51]. This 10-item scale measures global self-esteem, defined as the attitudes held about oneself. Participants rated items such as “I take a positive attitude toward myself” on a scale from 1 *(strongly disagree)* to 4 *(strongly agree)*. This scale has high internal consistency with previous estimates of .84 to .95 [52].

*Academic self-efficacy scale* [53]. This 5-item subscale, drawn from the Patterns of Adaptive Learning Scales, measures students’ perceptions of their academic competency. Participants rated items such as “even if the work is hard, I can learn it” from 1 *(not at all true)* to 5 *(very true)*. Midgley et al. produced an internal consistency estimate of .78, indicating acceptable reliability [53].

*Life orientation test-revised* [54]. This 6-item scale (plus four filler items) measures dispositional optimism. Participants rated items such as “in uncertain times I usually expected the best” from 1 *(I disagree a lot)* to 5 *(I agree a lot)*. The scale has produced an internal consistency estimate of .78, indicating acceptable reliability [54].

*Presence of meaning from the meaning in life scale* [38]. This 5-item subscale measures the perception that one’s life is meaningful. Participants rated items such as “my life has a clear sense of purpose” from 1 *(absolutely untrue)* to 7 *(absolutely true)*. Internal consistency estimates of .81 to .92 indicate good reliability [38].

*Academic buoyancy scale* [17]. This 4-item scale assesses students’ ability to deal with academic-related setbacks, challenges, and pressure. Participants rated items such as “I don’t let study stress get on top of me” from 1 (*strongly disagree*) to 7 (*strongly agree*). The scale has good reliability, with internal consistency estimates of .78 to .82 [17, 40].

*Adaptability scale* [40]. This 9-item scale measures the capacity to respond well to novel, changing, and/or uncertain circumstances. Participants rated items such as “I am able to think through a number of possible options to assist me in a new situation” on a scale from 1 (*strongly disagree*) to 7 (*strongly agree*). The scale has high reliability, with a previous internal consistency estimate of .90 [40].

#### Coping strategies

*Cognitive Emotion Regulation Questionnaire (CERQ)* [55]. This 36-item scale assesses nine cognitive coping strategies. Participants were asked to think about how they generally think when they experience negative events, and indicate how often they have thoughts such as “I think of what I can do best” on a scale from 1 *(almost never)* to 5 *(almost always)*. Each subscale has acceptable reliability with internal consistency estimates ranging from .66 to .83 [55].

*COPE inventory* [56]. This 60-item scale measures 15 strategies for coping with stress. Participants were instructed to indicate what they usually do when they experience stress. Participants rated items such as “I make a plan of action” on a scale from 1 (*I usually don’t do this at all*) to 4 (*I usually do this a lot*). Reliability of all subscales is generally acceptable, with median internal consistency estimates of around .75 [56].

#### Outcomes

*College adjustment questionnaire* [46]. This 14-item scale measures college functioning across three domains: educational, relational, and psychological. Participants indicated how accurately statements such as “I am succeeding academically” describe them, from 1 (*very inaccurate*) to 5 (*very accurate*). The scale has demonstrated good reliability in undergraduate samples, with internal consistency estimates ranging from .79 to .89 [46].

*Physical health questionnaire* [57]. This 14-item scale assesses sleep disturbances, headaches, respiratory infections, and digestive problems. Items such as “how often have you woken up during the night” were rated from 1 *(not at all)* to 7 *(all of the time)*. Internal consistency estimates indicate acceptable reliability, ranging from .79 to .88 for all subscales, except for the Respiratory Infections subscale which has previous estimates of .61 to .66 [57].

*Warwick-Edinburgh mental well-being scale* [47]. This 14-item scale measures subjective life satisfaction, and positive psychological functioning. Participants indicated how often they had experienced items such as “I’ve been feeling relaxed” over the last two weeks, on a scale from 1 *(none of the time)* to 5 *(all of the time)*. The scale has high internal consistency with estimates of .89 and .91 [47].

#### Control variables

*Esoteric Analogies Test (EAT)* [58]. This task measures fluid and crystallised intelligence in one total score. Participants were presented with a verbal analogy and instructed to identify which word out of four alternatives shared the same relationship with the target word. For example, “LIGHT is to DARK as HAPPY is to: GLAD, SAD*, GAY, EAGER”. This measure has acceptable reliability, with internal consistency estimates ranging from .64 to .76 [59, 60].

*Mini international personality item pool* [61]. This 20-item scale measures the Big 5 personality traits: Extraversion, Agreeableness, Conscientiousness, Neuroticism, and Intellect/Openness. Participants rated items such as “I have frequent mood swings” on a scale from 1 *(very inaccurate)* to 5 (*very accurate)*. All subscales possess acceptable internal consistencies of above .60, with some over .70 [61].

#### Procedure

Participants accessed the study using Qualtrics, an online survey system, and indicated their willingness to participate via a digital consent form. They first completed demographic questions (age, gender, financial variables), then the remaining measures were counterbalanced and participants were randomly assigned to one of four versions to reduce order effects.

## Results

### Descriptive statistics

#### Resilience resource measures

Descriptive statistics and reliability estimates (Cronbach’s alpha) for the resilience and related measures are presented in Table 1, and were consistent with previous research. Reliability estimates were acceptable to high, ranging from .71 to .91.

**Table 1:** Descriptive statistics and Cronbach’s alpha for resilience resource measures.

	M	SD	α
CD-RISC	2.49	.62	.86
Personal Competence	5.20	.84	.90
Acceptance of Self and Life	4.73	.88	.71
Academic Buoyancy	3.87	1.28	.83
Mental Toughness	4.82	.94	.88
Adaptability	4.97	.88	.88
Self-Esteem	2.74	.56	.90
Academic Self-Efficacy	3.72	.82	.90
Optimism	3.15	.75	.78
Presence of Meaning	4.35	1.35	.91

#### Coping measures

Descriptive statistics and Cronbach’s alphas for the coping measures are reported in Table 2. Mean scores and internal consistency coefficients were comparable to past research. Reliability estimates were acceptable to excellent, except for the Mental Disengagement subscale, with an estimate of .44 (possibly due to diversity in mental disengagement tactics) [56].

**Table 2:** Descriptive statistics and Cronbach’s alpha for coping measures.

	M	SD	*α*
**CERQ**			
Refocus on Planning	12.95	3.24	.80
Acceptance	13.50	2.97	.71
Positive Reappraisal	12.34	3.91	.85
Putting into Perspective	12.68	3.75	.74
Positive Refocusing	9.53	3.47	.84
Self-Blame	12.19	3.36	.79
Rumination	13.58	3.33	.72
Catastrophising	9.14	3.22	.72
Other-Blame	7.80	2.49	.74
**COPE Inventory**			
Positive Reinterpretation	11.07	2.76	.82
Planning	11.36	2.86	.87
Acceptance	11.09	2.51	.76
Active Coping	10.87	2.62	.80
Suppression of Competing Activities	10.07	2.36	.61
Restraint	9.41	2.30	.67
Mental Disengagement	10.21	2.53	.44
Denial	6.07	2.37	.79
Behavioural Disengagement	6.81	2.43	.79
Religious Coping	6.43	3.80	.96
Substance Use	6.05	3.30	.97
Humour	9.00	3.43	.91
Venting	10.50	3.21	.84
Instrumental Support Seeking	11.09	3.40	.87
Emotional Support Seeking	10.96	3.73	.93

#### Outcome and control measures

Table 3 presents the mean scores and reliability coefficients for the outcome and control variables, which were consistent with previous research. 70.6% of the sample reported they live with parents.

**Table 3:** Descriptive statistics and reliability coefficients (Cronbach’s Alpha) for outcome and control measures.

	M	SD	α
**Outcome Measures**			
Warwick-Edinburgh Mental Well-being Scale	45.41	9.78	.93
College Adjustment Questionnaire	40.80	9.46	.88
Physical Health Questionnaire	44.83	12.85	.82
**Personality Measure**			
Mini International Personality Item Pool			
Extraversion	11.62	3.91	.83
Agreeableness	16.17	3.17	.80
Conscientiousness	13.29	3.23	.69
Neuroticism	12.76	3.34	.69
Intellect	15.08	3.01	.71
**Intelligence Measure**			
Esoteric Analogies Test			
Accuracy	69.72	15.11	.66
**Financial Measures**			
Weekly employment hours	9.78	9.32	-
Subjective social class	61.09	18.59	-
Perceived financial comfort	53.51	21.23	-

### Aim 1

Table 4 summarises Pearson correlations and results of an EFA (Maximum Likelihood with Promax rotation) performed on the resilience resource measures via SPSS (v24). There were moderate to strong positive correlations between all measures (*r* = .26 to .72, *p* < .01). Overall, the positive manifold suggests these measures converge, supporting Hypothesis 1. This was supported by the EFA, where the latent root criterion (eigenvalues greater than one) and scree plot clearly indicated one factor (see S1 Fig). This factor explained 50.82% of the common variance (KMO = .91). For comparison, we tested alternative models, constraining the solution to two and three factors. The two factor solution explained 56.54% of variance, with the second factor uniquely accounting for 5.25%. The factors shared a high correlation of .71. The three factor solution accounted for 61.46% of variance; however, factors 2 and 3 were each only defined by two indicators, where three is the suggested minimum for meaningful interpretation [62]. The three factors were also highly correlated, ranging from .62 to .75. Given the scree plot, eigenvalues greater than one, the small amount of variance explained by the additional factors, and high correlations between them, the one-factor model was retained. All factor loadings were high and positive, ranging from .51 to .85. Communalities were also generally high, ranging from .26 to .72. The one-factor solution was consistent with correlation patterns and supported Hypothesis 1 that an overarching factor accounts for shared variance between these measures. A composite score was created using the Bartlett method [see 63].

**Table 4:** Correlations, factor loadings, and communalities for resilience resource measures.

	2	3	4	5	6	7	8	9	10	Factor Loadings	*h*2
1 CD-RISC	.71	.62	.56	.63	.67	.57	.44	.43	.43	.82	.67
2 Personal Competence		.72	.48	.72	.67	.55	.43	.40	.43	.85	.72
3 Acceptance of Self and Life			.53	.61	.54	.62	.30	.49	.55	.80	.64
4 Academic Buoyancy				.45	.48	.46	.30	.41	.29	.62	.38
5 Mental Toughness					.57	.56	.48	.46	.51	.79	.63
6 Adaptability						.56	.42	.46	.33	.75	.57
7 Self-Esteem							.35	.65	.49	.73	.54
8 Academic Self-Efficacy								.31	.26	.51	.26
9 Optimism									.44	.60	.36
10 Presence of Meaning										.57	.33

*Note*. All correlations are significant at *p* < .01.

### Aims 2 and 3

A path analysis using the Maximum Likelihood method was conducted to examine aims 2 and 3. That is, whether resilience resources predict outcomes controlling for covariates, and whether coping responses mediate these relationships. An EFA of the coping measures was first run to determine the underlying latent factors for use in the path model. Given that determining factorial convergence of the coping measures is outside of the focus, the detailed results and interpretations are presented in S1 Appendix. This resulted in five factors accounting for 49.07% of the common variance. Factor 1 was labelled *Problem-focused Coping*, defined by active coping, planning, and suppression of competing activities. Factor 2 was labelled *Positive Thinking*, defined by putting into perspective, positively reappraising and refocusing. Factor 3 was labelled *Support-seeking*, defined by venting, and seeking emotional and instrumental support. Factor 4 was labelled *Avoidant Coping*, defined by disengaging from and denying the problem. Factor 5 was labelled *Maladaptive Coping*, defined by less-adaptive self-focused cognitions and behaviours. The Bartlett method was used to create composite factorial scores for each factor, for use in subsequent analyses.

#### Correlations

Table 5 presents correlations between all variables in the path model. The resilience resources factor shared significant correlations with all coping factors, in the expected directions. It also correlated significantly with each of the three outcome variables, all personality dimensions, age, gender, financial comfort, and social class.

**Table 5:** Correlations between all variables in the path model.

	2	3	4	5	6	7	8	9	10	11	12	13	14	15	16	17	18	19	20	21
1 Resilience resources factor	.60**	.57**	.25**	-.41**	-.21**	.05	.32**	.19**	.33**	-.50**	.30**	.18**	-.11*	.01	.27**	.13*	.09	.57**	.71**	-.25**
**Coping**																				
2 Problem-focused	1	.41**	.36**	-.15**	.10	.02	.10	.09	.24**	-.22**	.24**	.17**	-.10	-.07	.13*	-.00	.01	.29**	.26**	-.06
3 Positive thinking		1	.11	-.11	-.04	-.16**	.18**	.11*	.08	-.44**	.13*	.04	-.17**	.01	.14*	.09	.07	.31**	.43**	-.15*
4 Support-seeking			1	-.04	.02	-.05	.38**	.31**	.04	.08	.09	.06	.16**	-.17**	.11	.14*	.06	.26**	.31**	-.01
5 Avoidant				1	.22**	-.23*	-.13*	-.29**	-.27**	.24**	-.03	-.00	.05	-.10	-.17**	-.14**	-.01	-.31**	-.30**	.37**
6 Maladaptive					1	-.01	-.15**	-.06	-.19**	.36**	.19**	-.09	-.06	-.03	-.03	-.01	.00	-.25**	-.26**	.25**
**Intelligence**																				
7 EAT accuracy						1	-.11	.13*	.03	-.02	.17**	.14*	-.05	.05	.05	-.03	-.01	.00	-.05	-.10
**Personality**																				
8 Extraversion							1	.32**	-.08	-.14**	.17**	.07	.01	-.12*	.10	.19**	.20**	.31**	.29**	.03
9 Agreeableness								1	.17**	-.04	.27**	.02	.06	.04	.17**	.10	.06	.14*	.20**	-.04
10 Conscientiousness									1	-.16**	.00	.15**	.06	.05	.12*	.02	.01	.21**	.23**	-.14*
11 Neuroticism										1	-.03	-.01	.29**	-.08	-.09	-.11	.05	-.37**	-.46**	.42**
12 Intellect											1	.08	-.11	-.07	.06	-.01	.03	.09	.20**	.01
**Demographics**																				
13 Age												1	.01	-.23**	.01	-.16**	.32**	.03	.04	.02
14 Gender													1	-.02	-.04	-.05	.03	-.11	-.09	.19**
15 Live with parents														1	.00	-.03	-.03	.03	.10	-.14*
16 Financial comfort															1	.35**	.11*	.31**	.30**	-.19**
17 Social status																1	-.02	.27**	.23**	-.06
18 Hours work																	1	.11	-.00	.11*
**Outcomes**																				
19 Adjustment																		1	.59**	-.24**
20 Mental well-being																			1	-.40** 1
21 Somatic health symptoms																				

Note. * p < .05;

** p < .01.

All five coping factors demonstrated moderate relationships with adjustment and well-being. Only the positive thinking, avoidant, and maladaptive coping factors shared relationships with somatic health symptoms. Personality dimensions demonstrated some moderate relationships with the coping factors and outcome variables. The outcome variables demonstrated moderate intercorrelations with each other, and in the expected direction. Adjustment and well-being were generally unrelated to demographics, with the exception of financial-related variables. Somatic health symptoms additionally shared small correlations with living situation, work hours, and gender. Intelligence, age, and gender were generally unrelated or weakly related to the variables of interest.

#### Path analysis

All possible regression paths were first built into the path model. Consistent with the results of the EFA, the coping factors were allowed to correlate with each other, as were the outcome variables. All control variables were retained in the model to enable strong conclusions about the role of resilience resources in predicting outcomes. With the exception of the TLI, this model had near perfect fit: χ^2^_2_ = 0.91, *p* = .40, RMSEA = .00 (90% CI: .00, .11), CFI = 1.00, GFI = 1.00, TLI = 1.01. However, many paths were non-significant, pointing to the model being over-fitted. This interpretation is consistent with a TLI > 1 [see 64]. To rectify this, all non-significant correlations and regression paths (*p* > .05) were constrained to zero. The final model returned excellent fit without any indication of being over-fitted: χ^2^_129_ = 1.09, *p* = .22, RMSEA = .02 (90% CI: .00, .03), CFI = .99, GFI = .96, TLI = .99. The model accounted for 58.1% variance in well-being, 38.7% in adjustment, and 27.6% in somatic health symptoms. For comparison, the model was also run excluding all control variables, with the resilience factor, coping factors, and outcome variables kept the same. The results are presented in S2 Appendix. Overall, a similar pattern of relationships emerged.

#### Direct effects

**Fig 2:**
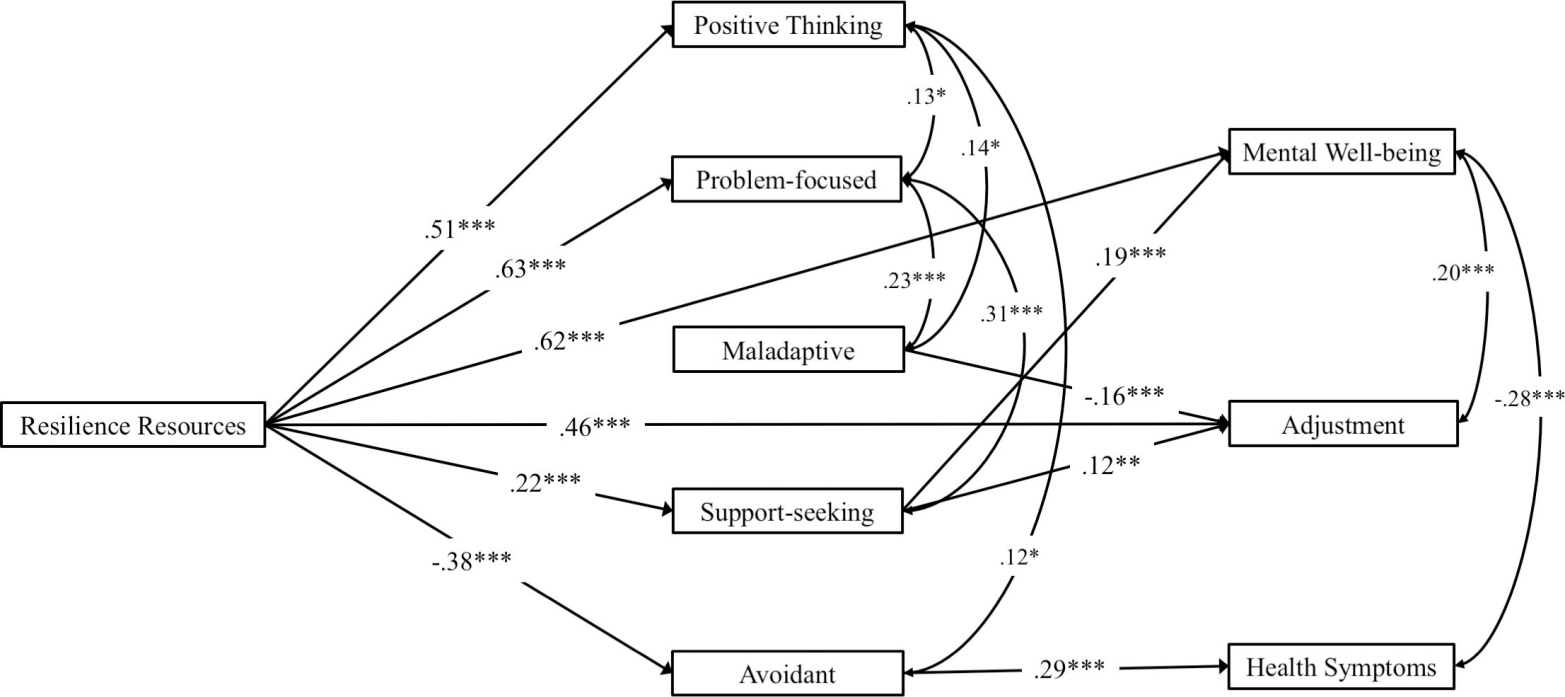
Significant direct effects for variables relevant to the hypotheses.

For ease of interpretability, Fig 2 depicts statistically significant paths for variables central to the hypotheses only. The full results of the analysis are presented in S1 Table. Accounting for all other variables in the model, the resilience resources factor strongly predicted well-being and adjustment (*β =* .62 and .49, *p* < .001), supporting Hypotheses 2.1 and 2.2. This illustrates that personal resilience attributes have a significant impact on the positive functioning of university students, even after accounting for financial factors, employment, and living situation. The resilience factor also had a positive direct effect on problem-focused (*β =* .63, *p* < .001), positive thinking (*β =* .51, *p* < .001), and support-seeking (*β* = .22, *p* < .001); and a negative effect on avoidant coping (*β =* -.38, *p* < .001).

Support-seeking positively predicted well-being (*β* = .19, *p* < .001). Adjustment was weakly predicted by support-seeking (*β =* .12, *p* < .01) and negatively by maladaptive coping (*β* = -.16, *p* < .001). Avoidant coping moderately predicted greater somatic health symptoms (*β* = .29, *p* < .001). These direct effects suggest that coping factors may act as mediators between resilience resources and outcome variables, which was investigated through indirect effects (see next section).

With regards to control variables, mental well-being was additionally predicted by lower Neuroticism, living with parents, lower intelligence, and higher social status. Adjustment was only weakly predicted by financial comfort and social status. Lower financial comfort, and higher scores on Extraversion and Neuroticism predicted greater reporting of somatic symptoms. Problem-focused coping was predicted by introversion, whereas positive thinking coping was predicted by lower scores on Neuroticism, Conscientiousness, and intelligence. Support-seeking was negatively predicted by gender and living with parents, such that males and those who live out of home appear to seek more support to cope with stressors. Higher scores on Extraversion, Neuroticism, and Agreeableness also predicted greater support-seeking. Avoidant coping was predicted by lower Agreeableness and intelligence, and greater Intellect. Maladaptive coping was predicted by lower Extraversion and Conscientiousness, and higher Neuroticism and Intellect. It was also negatively predicted by gender, such that males appeared to engage in more maladaptive coping.

#### Indirect and total effects

Hypothesis 3, that coping styles mediate the relationships between resilience resources and outcome variables, was tested by the indirect effects. Resilience resources had a significant indirect effect on adjustment (*β =* .03, *p* < .01) and somatic health symptoms (*β =* -.13, *p* < .001). As resilience resources did not have a significant direct effect on somatic health symptoms, this indicates that the relationship was fully mediated by avoidant coping, the only coping factor which predicted this outcome. Resilience resources had both a significant direct and indirect effect on adjustment, indicating partial mediation by support-seeking. Overall, these findings provide some support for Hypothesis 3, in that resilience resources influence outcomes via their impact on coping responses, specifically for physical health and university adjustment. S1 Table summarises all direct and indirect effects, with variables in order, from largest to smallest, of their absolute total effect.

## Discussion

Varying conceptualisations (*trait*, *process*, *outcome*) and consequently, operationalisations, exist in the resilience literature. This research is the first to empirically determine a possible synergy between these approaches in an integrative model and clarify our understanding of resilience and its measurement model. The first aim targeted *trait* aspects of resilience through examining the convergence and factorial structure of self-report measures of resilience and related constructs. Based on the integration of resilience process theories, the second and third aims examined the predictive validity of resilience-related measures on markers of positive adaptation; and whether these relationships were mediated by the way people respond to stressors with varying coping strategies. This was examined in the tertiary academic environment, capturing relevant positive adaptation metrics. Overall, we found support for the proposed mechanisms underlying resilience in an academic context.

### Resilience resources: Evidence of protective factors

Resilience research has commonly employed self-report questionnaires to measure the construct. To investigate their construct validity, we examined a selection of widely used scales purporting to measure resilience and related personal resources. The EFA provided robust support for Hypothesis 1, that these measures would positively converge onto a latent factor. This illustrates that self-report measures tap into the personal attributes contributing to one’s resilience, and that robust individual differences exist.

Resilience is argued to be dependent on the situation, but there appear to be stable individual differences which may generalise across contexts. Measures included in this study were three resilience scales that have been employed in different domains: CD-RISC (clinical, sport, defence), Wagnild & Young’s Resilience Scale (at-risk populations, general population), and the Academic Buoyancy Scale (education). The third proposes to measure a context-specific resilience to everyday academic pressures [17]. This measure moderately correlated with other resilience scales. The unique residual variance in academic buoyancy not accounted for by the latent factor, is consistent with Martin and Marsh’s claim that buoyancy is a necessary but not sufficient component of resilience [65].

We also captured mental toughness, self-esteem, academic efficacy, optimism, presence of meaning, and adaptability. The three resilience scales shared strong relationships and convergence with mental toughness and adaptability which may raise questions of their discriminant validity, as there are purported conceptual distinctions between resilience and these constructs. Mental toughness refers to a personal attribute, unlike resilience which is best thought of as a process involving both internal and external factors [27]. Adaptability is similarly a personal characteristic relating specifically to situations of uncertainty and novelty, whereas resilience relates to stressors or adversity more broadly [40]. However, the results illustrate that these self-report scales are unable to capture this distinction fully.

### Mechanisms involved in the resilience process

Several models have been proposed depicting the mechanisms involved in the resilience process. We sought to examine a possible synergy between these models and adapted their ideas to the academic context. We examined the impact of personal resilience resources (or protective factors) on markers of positive adaptation, and the mediatory role of how one responds through coping strategies.

Firstly, resilience resources were positively related to positive thinking, problem-focused-coping, and support-seeking, and negatively related to avoidant coping. Previous research on university students shows similar findings; whereby academic resilience was positively related to problem-focused and positive coping styles, and negatively related to avoidant strategies [66]. However, Meneghel et al. [66] found a negative relationship between socially-oriented coping strategies and academic resilience. This difference may be, in part, due to the difference in relative weightings of emotional versus instrumental strategies on the broad factor. In this study, the support-seeking factor was defined more strongly by instrumental support than venting. Turning to others for advice plausibly has a more positive and proactive impact on overcoming challenges than venting.

Second, the resilience resources factor was the strongest predictor of mental well-being, supporting Hypothesis 2.1. This finding is consistent with meta-analyses demonstrating robust relationships between resilience and well-being measures [67]. Support-seeking also predicted mental well-being, though did not act as a mediator. Of note, is the high correlation between mental well-being and the resilience composite factor, which was likely inflated due to shared method variance. The cross-sectional nature of this study precludes interpretations about the direction of this relationship. Thus, longitudinal studies are needed to examine whether resilient attributes enhance future well-being, or greater well-being enhances resilience. Previous longitudinal research on the Big Five personality traits and subjective well-being suggests a reciprocal relationship [68]. Future studies should take a similar approach to delineate the relationship between self-reported resilience and aspects of well-being.

Third, the resilience resources composite was also the strongest predictor of university adjustment, supporting Hypothesis 2.2. This relationship was partially mediated; such that internally resilient individuals appeared to have a greater tendency to cope through seeking support which in turn facilitated better adjustment. Support-seeking and lesser use of maladaptive strategies also enhanced adjustment directly. The mediatory relationship is consistent with similar previous findings showing that optimism, control, and self-esteem enhance adjustment through greater support-seeking [69]. Our finding sheds light on the mechanisms through which university students can more positively adapt in the face of stressors.

Finally, resilience resources indirectly predicted somatic health symptoms through avoidant coping. This is consistent with personality and health research suggesting that such relationships exist due to the influence personality factors have on behaviours, which in turn affect health [70, 71]. Thus, possessing greater resilient attributes is seemingly beneficial for the physical health of students by reducing the tendency to employ avoidant coping strategies.

Importantly, these relationships existed after controlling for a number of individual differences and situational factors, which are briefly discussed below. Contrary to what might be expected, those *lower* on extraversion engaged in more problem-focused strategies, as well as more maladaptive strategies. However, these predictions were relatively small and should be interpreted with caution. Those *high* on neuroticism employed less positive thinking and more maladaptive strategies such as substance use and rumination, whilst the opposite was the case for conscientiousness. This is consistent with expectations given that those high in neuroticism experience more intense negative emotions, thus might be less likely to engage in positive emotional strategies; and those high in conscientiousness display better self-discipline and emotion-regulation [72]. Higher scores on the intelligence measure also predicted positive thinking and less use of avoidant strategies. Consistent with the findings of a meta-analysis, higher extraversion, agreeableness, and neuroticism predicted greater support-seeking [72]. Males and those living with their parents also reported seeking support more. Intellect/openness predicted greater use of both avoidant and maladaptive coping. Those high on intellect/openness tend to be more imaginative, wishful, and open to new perspectives which might explain greater use of avoidant strategies which involve disengagement and fantasising [72]. Similar reasons might explain the relationship with the maladaptive coping factor, which was defined in part by rumination and acceptance. Finally, more agreeable people appeared to use less avoidant coping strategies including disengagement and denial, which is consistent with the notion that agreeableness is associated with greater acceptance coping [72].

With regards to the outcome variables, financial comfort and subjective social status were also significant predictors of adjustment, consistent with previous research [73]. Previous findings have indicated that those scoring lower on Neuroticism, and higher on Openness and Conscientiousness, experience better overall adjustment [74]. Zero-order correlations in this study showed similar relationships; however, once all variables were included in the model, personality dimensions lost significance, and the resilience factor strongly predicted adjustment beyond all other variables. This highlights the important role which resilience resources play in enhancing adjustment beyond other known predictors.

Somatic health symptoms were most strongly predicted by Neuroticism. Previous research has demonstrated Neuroticism to be positively related to hypochondriasis [75], and negatively related to practicing health-promoting behaviours [76], which may provide explanations for this relationship. However, given that somatic symptoms were self-reported, this can only be interpreted as an association with tendency to report complaints, rather than objective physical symptoms.

### Towards an integrated conceptualisation

Resilience literature has debated whether to conceptualise resilience as a trait, process, or outcome. However, we propose that none of these perspectives are sufficient on their own. Whilst there are stable (trait-like) individual differences in resilient attributes, we must contextualise these characteristics within the overall resilience process in order to examine the role that these traits play in various contexts. Further, whilst resilience may be inferred based on a positive outcome following the process, such an outcome of ‘resilience’ cannot logically occur in isolation of the process through which this outcome arises. Thus, we propose resilience may be best examined with a view of synergy between traits, process, and outcomes. Our findings suggest that individual differences influence the way one appraises and responds in the face of a stressor leading to a particular outcome. Both the selection and usefulness of coping strategies will vary situationally. For example, emotion-focused strategies are typically considered more adaptive in situations that are beyond our control; whereas in situations which we can control, problem-focused strategies may be more beneficial, thus leading to different outcomes in different situations. This perspective reconciles and aligns with the varying definitions outlined in the introduction. For instance, Zautra and Reich’s [5] definition of resilience as recovery, sustainability, and growth as seen through coping and adaptation highlights the role of the dynamic coping process as well as the integral role of adaptive outcomes, without which resilience cannot exist. Similarly, it fits with the APA’s [4] definition of resilience as a process *and* outcome. Finally, Fletcher and Sarkar’s [3] definition emphasises the protective role of mental processes and personal assets, consistent with findings that the resilience resources factor influenced coping behaviour and adaptive outcomes.

### Limitations and future directions

There are a number of limitations to consider when interpreting these findings. Firstly, resilience research is most meaningful and interpretable in the context of a stressor or adverse event. The current study sought to investigate the process of resilience when facing challenges at university. Thus, a homogeneous university sample was used, and it was assumed that they were subject to stressors during their university experience. Future studies should take this research one step further by quantifying stress or adversity to examine the buffering effect that protective resilience resources have against the potential negative impacts of stressors on outcomes.

Further research is also needed to assess the extent to which these findings generalise to other samples, both in academic and other contexts. The goal of this research is to develop a general model of resilience that can be adapted to different contexts. It might be expected that the specific findings regarding outcomes and coping strategies would generalise to other university student samples; however, the resilience literature advocates for context-specific investigations of resilience. Thus, future studies should examine the extent to which the general mechanisms (i.e., resilience resources, coping responses, adaptive outcomes) apply across other contexts with context-specific outcomes. It would be expected that the specific resilience resources and coping responses that lead to adaptive outcomes would differ between contexts. This is an important avenue for the establishment of an externally valid general resilience model that can be adapted to different domains.

Additionally, the cross-sectional design of this study prevents our capacity to make causal inferences. The designation of measures as predictors or outcomes were made on theoretical grounds; however, the direction of relationships cannot be inferred from the results. Longitudinal research is needed to validate the novel model. Such research might track students over the course of their degree and beyond, allowing an examination of the relationship between resilience and future objective outcomes such as academic performance, drop-out rates, enrolment in postgraduate study, or securing employment. Nevertheless, this study extends upon previous resilience research in the academic domain which has typically conceptualised resilience as a capacity, or has indirectly inferred resilience based on academic success rather than taking a holistic approach to capturing the process in its entirety.

Thirdly, the focus of this study was on the individual-level differences in mental resilience. However, it is also acknowledged that the resilience process encompasses societal and cultural factors [77]. Future studies should extend this research to include social, cultural, and environmental differences and examine their role in the resilience process.

### Practical implications and conclusions

In sum, this study provides empirical support to existing theories of resilience, shedding light on the relationships between individual differences and coping mechanisms associated with resilience and positive outcomes. There is growing interest in resilience interventions in the higher education context. Common interventions consist of cognitive-behavioural and mindfulness-based stress reduction strategies [see 78]. However, emotion-based strategies form just one part of the picture. Interventions should also target skills and strategies for coping with challenges (e.g., seeking social support, reducing avoidance and disengagement) and provide appropriate resources such as programs which facilitate social connection (e.g., mentoring, peer support groups) and accessible student support. Additionally, having identified the attributes of individuals who appear to cope more adaptively and experience better overall well-being, these findings might be used to identify students at-risk of increased vulnerability to stress to provide them with appropriate resources and support.

## Supporting information

S1 Fig Scree plot. (TIF)

S1 Appendix Factor analysis of coping measures. (DOCX)

S2 Appendix Path model without control variables. (DOCX)

S1 Table Significant direct, indirect, total effects, and R^2^. (DOCX)
