# Supplementary figures and images for: An integrative process model of resilience in an academic context: Resilience resources, coping strategies, and positive adaptation
Source: PLoS One. 2021 Feb 2;16(2):e0246000. doi: 10.1371/journal.pone.0246000 (PMC7853478; doi:10.1371/journal.pone.0246000)

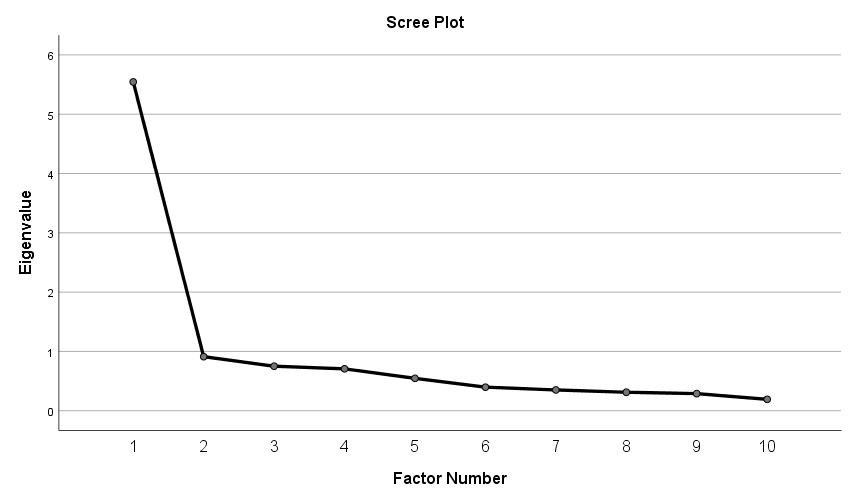

Supplement: S1 Fig — (TIF) [file pone.0246000.s001.tif]
