# Supplementary material for: An integrative process model of resilience in an academic context: Resilience resources, coping strategies, and positive adaptation
Source: PLoS One. 2021 Feb 2;16(2):e0246000. doi: 10.1371/journal.pone.0246000 (PMC7853478; doi:10.1371/journal.pone.0246000)
Supplement: S1 Appendix — (DOCX) [file pone.0246000.s002.docx]

**S1 Appendix.**

**Factor Analysis of Coping Measures**

An EFA (Principal Axis Factoring with Promax rotation) was run on all subscales from the COPE Inventory and CERQ. Four criteria were considered in determining the number of factors to extract: the latent root criterion, the scree test, amount of common variance accounted for, and the meaningfulness of the rotated factors. The latent root criterion indicated up to seven factors, explaining 55.41% of the common variance. However, factors 6 and 7 were each defined by only two variables with factor loadings over 0.3 and uniquely explained 2.99% and 2.21%. Examining the scree plot and meaningfulness of factors indicated the possibility of five factors. A second EFA constraining the solution to five factors accounted for 49.07% of the common variance. Two subscales, Religious Coping and Substance Use, had low communalities (.14). Religious Coping did not load strongly onto any factor. Catastrophising and Acceptance (CERQ) had loadings over |.30| on two factors, and the Venting subscale on three. The results of this EFA are presented in Table A1.

**Table A1. Factor Loadings, Communalities, Eigenvalues, % Variance for Coping Measures**

|  | 1 | 2 | 3 | 4 | 5 | *h*2 |
| --- | --- | --- | --- | --- | --- | --- |
| Active coping (COPE) | **.91** | -.08 | -.01 | -.10 | -.06 | .79 |
| Planning (COPE) | **.85** | -.04 | .01 | -.02 | -.06 | .70 |
| Refocus on planning (CERQ) | **.62** | .28 | -.09 | -.12 | .15 | .67 |
| Suppression of competing activities (COPE) | **.60** | .03 | .01 | .11 | .05 | .35 |
| Religious coping (COPE) | .29 | .01 | .03 | .28 | -.24 | .14 |
| Putting into perspective (CERQ) | -.11 | **.77** | .03 | -.04 | .06 | .53 |
| Positive reappraisal (CERQ) | .28 | **.65** | .00 | -.06 | -.05 | .70 |
| Refocusing on positives (CERQ) | .09 | **.56** | .03 | .27 | -.17 | .41 |
| Positive reinterpretation (COPE) | .31 | **.54** | .22 | .01 | -.15 | .69 |
| Acceptance (COPE) | .10 | **.51** | -.08 | .01 | .25 | .36 |
| Humour (COPE) | -.24 | **.46** | .08 | .21 | .09 | .21 |
| Emotional support-seeking (COPE) | -.10 | .12 | **.99** | -.05 | -.06 | .97 |
| Instrumental support-seeking (COPE) | .09 | .13 | **.75** | -.02 | -.04 | .69 |
| Venting (COPE) | .02 | **-.31** | **.62** | .07 | **.30** | .55 |
| Behavioural disengagement (COPE) | -.32 | .10 | .00 | **.68** | -.03 | .60 |
| Denial (COPE) | .00 | .04 | -.10 | .**66** | -.07 | .43 |
| Catastrophising (CERQ) | .29 | -.28 | -.03 | **.54** | **.35** | .62 |
| Mental disengagement (COPE) | -.20 | .21 | .05 | **.46** | .11 | .30 |
| Restraint (COPE) | .28 | .22 | -.08 | **.42** | -.03 | .27 |
| Other-blame (CERQ) | .15 | .00 | .09 | **.41** | .08 | .22 |
| Rumination (CERQ) | .14 | .07 | .21 | -.03 | **.66** | .55 |
| Acceptance (CERQ) | -.05 | **.50** | -.17 | -.02 | **.60** | .55 |
| Self-blame (CERQ) | -.06 | -.04 | -.03 | .01 | **.58** | .34 |
| Substance use (COPE) | -.12 | .05 | .02 | .13 | **.31** | .14 |
| Eigenvalue  % of Variance | 5.39  20.91% | 3.13  10.74% | 2.28  7.91% | 1.78  5.66% | 1.47  3.84% |  |
| *Factor Intercorrelations* |  |  |  |  |  |  |
| 1 |  | .48 | .37 | -.19 | .13 |  |
| 2 |  | 1 | .15 | -.13 | -.05 |  |
| 3 |  |  | 1 | -.05 | .01 |  |
| 4 |  |  |  | 1 | .31 |  |
| 5 |  |  |  |  | 1 |  |

*Note.* Factor loadings over |.30| are bolded.
