# Supplementary material for: An integrative process model of resilience in an academic context: Resilience resources, coping strategies, and positive adaptation
Source: PLoS One. 2021 Feb 2;16(2):e0246000. doi: 10.1371/journal.pone.0246000 (PMC7853478; doi:10.1371/journal.pone.0246000)
Supplement: S2 Appendix — (DOCX) [file pone.0246000.s003.docx]

**S2 Appendix. Path Model Without Control Variables.**

The model was first run with all possible regression paths built into the model. Coping factors were allowed to correlate with each other, as were outcome variables. The model had zero degrees of freedom, indicating the model was over-fitted. To rectify this, all non-significant paths were constrained to zero, increasing the degrees of freedom (Byrne, 2001). This model returned excellent fit with no indication of being over-fitted: *χ*^2^ _15_ = 1.48, *p* = .10, RMSEA = .04 (90% CI: .00, .07), CFI = .99, GFI = .98, TLI = .98. Importantly, all regression paths maintained significance. Table A presents the results for both the over-fitted and constrained models, for comparison. Overall, a similar pattern of results emerged to the full model with control variables.

Table A

*Direct Effects, Indirect Effects and R^2^ Values for the Unconstrained and Constrained Models*

| Endogenous Variable  Predictor | Unconstrained Model | | | Constrained Model | | |
| --- | --- | --- | --- | --- | --- | --- |
|  | Direct | Indirect | R^2^ | Direct | Indirect | R^2^ |
| Mental well-being |  |  | .55 |  |  | .54 |
| Resilience | .68*** | .03 |  | .70*** | .00 |  |
| Problem-focused | -.14** |  |  | -.11* |  |  |
| Support seeking | .19*** |  |  | .19*** |  |  |
| Positive thinking | .07 |  |  |  |  |  |
| Avoidant | -01 |  |  |  |  |  |
| Maladaptive | -.10* |  |  | -.11** |  |  |
| Adjustment |  |  | .37 |  |  | .36 |
| Resilience | .51*** | .06*** |  | .50*** | .07*** |  |
| Problem-focused | -.09 |  |  |  |  |  |
| Support seeking | .17*** |  |  | .14*** |  |  |
| Positive thinking | .02 |  |  |  |  |  |
| Avoidant | -.07 |  |  |  |  |  |
| Maladaptive | -.12* |  |  | -.15** |  |  |
| Physical health symptoms |  |  | .18 |  |  | .17 |
| Resilience | -.07 | -.18*** |  | - | -.17*** |  |
| Problem-focused | .05 |  |  |  |  |  |
| Support seeking | .01 |  |  |  |  |  |
| Positive thinking | -09 |  |  |  |  |  |
| Avoidant | .31*** |  |  | .33*** |  |  |
| Maladaptive | .16** |  |  | .18** |  |  |
| Problem-focused |  |  | .36 |  |  | .36 |
| Resilience | .60*** |  |  | .60*** |  |  |
| Support seeking |  |  | .06 |  |  | .06 |
| Resilience | .25*** |  |  | .25*** |  |  |
| Positive thinking |  |  | .33 |  |  | .33 |
| Resilience | .57*** |  |  | .57*** |  |  |
| Avoidant |  |  | .17 |  |  | .17 |
| Resilience | -.41*** |  |  | -.41*** |  |  |
| Maladaptive |  |  | .04 |  |  | .04 |
| Resilience | -.21*** |  |  | -.21*** |  |  |

*Note.* Direct and indirect effects are reported in standardised form (*β*).

**p*<.05; ***p*<.01; ****p*<.001.
