# Supplementary material for: An integrative process model of resilience in an academic context: Resilience resources, coping strategies, and positive adaptation
Source: PLoS One. 2021 Feb 2;16(2):e0246000. doi: 10.1371/journal.pone.0246000 (PMC7853478; doi:10.1371/journal.pone.0246000)
Supplement: S1 Table — (DOCX) [file pone.0246000.s004.docx]

**S1 Table. Significant Direct, Indirect, Total Effects, and R^2^.**

| Endogenous Variable  Predictor | Direct Effect | | | Indirect Effect | Total Effect | R^2^ |
| --- | --- | --- | --- | --- | --- | --- |
|  | B | SE | *β* |  |  |  |
| Mental well-being |  |  |  |  |  | .58 |
| Resilience | 5.77 | .47 | .62*** | -.01 | .61** |  |
| Support-seeking | 1.83 | .39 | .19*** | — | .19*** |  |
| Neuroticism | -.37 | .13 | -.13** | .01 | -.12* |  |
| Social status | .05 | .02 | .10** | — | .10* |  |
| Intelligence | -.05 | .02 | -.08* | — | -.08* |  |
| Extraversion | — | — | — | .07*** | .07*** |  |
| Living with parents | 1.92 | .74 | .09** | -.02* | .07 |  |
| Agreeableness | — | — | — | .04*** | .04*** |  |
| Gender | — | — | — | .03** | .03** |  |
| Adjustment |  |  |  |  |  | .39 |
| Resilience | 4.15 | .44 | .46*** | .03** | .48** |  |
| Maladaptive | -1.29 | .38 | -.16*** | — | -.16*** |  |
| Social status | .08 | .02 | .15** | — | .15* |  |
| Support-seeking | 1.12 | .43 | .12** | — | .12** |  |
| Financial comfort | .05 | .02 | .12* | — | .12* |  |
| Extraversion | — | — | — | .05*** | .05*** |  |
| Intellect | — | — | — | -.03*** | -.03*** |  |
| Gender | — | — | — | .03** | .03** |  |
| Neuroticism | — | — | — | -.03* | -.03* |  |
| Conscientiousness | — | — | — | .02** | .02** |  |
| Agreeableness | — | — | — | .02** | .02* |  |
| Living with parents | — | — | — | -.01* | -.01* |  |
| Somatic health symptoms |  |  |  |  |  | .28 |
| Neuroticism | 1.29 | .20 | .34*** | -.01 | .33*** |  |
| Avoidant coping | 3.24 | .55 | .29*** | — | .29*** |  |
| Resilience | — | — | — | -.13*** | -.13*** |  |
| Financial comfort | -.07 | .03 | -.12* | — | -.12* |  |
| Extraversion | .45 | .17 | .14** | -.02 | .12* |  |
| Agreeableness | — | — | — | -.08*** | -.08*** |  |
| Intelligence | — | — | — | -.06*** | -.06*** |  |
| Intellect | — | — | — | .05** | .05** |  |
| Problem-focused coping |  |  |  |  |  | .37 |
| Resilience | .64 | .05 | .63*** | — | .63*** |  |
| Extraversion | -.03 | .01 | -.10* | — | -.10 |  |
| Positive thinking |  |  |  |  |  | .41 |
| Resilience | .53 | .06 | .51*** | — | .51*** |  |
| Neuroticism | -.07 | .01 | -.22*** | — | -.22*** |  |
| Intelligence | -.01 | .00 | -.18*** | — | -.18*** |  |
| Conscientiousness | -.04 | .02 | -.12** | — | -.12* |  |
| Support seeking |  |  |  |  |  | .25 |
| Extraversion | .07 | .01 | .26*** | — | .26*** |  |
| Resilience | .21 | .06 | .22*** | — | .22** |  |
| Agreeableness | .06 | .02 | .19*** | — | .19*** |  |
| Neuroticism | .05 | .02 | .16** | — | .16** |  |
| Gender | .32 | .11 | .14** | — | .14* |  |
| Living with parents | -.20 | .10 | -.09* | — | -.09* |  |
| Avoidant coping |  |  |  |  |  | .28 |
| Resilience | -.41 | .06 | -.38*** | — | -.38*** |  |
| Agreeableness | -.08 | .02 | -.23*** | — | -.23*** |  |
| Intelligence | -.02 | .00 | -.21*** | — | -.21*** |  |
| Intellect | .07 | .02 | .19*** | — | .19** |  |
| Maladaptive coping |  |  |  |  |  | .20 |
| Neuroticism | .11 | .02 | .34*** | — | .34*** |  |
| Intellect | .07 | .02 | .19*** | — | .19*** |  |
| Conscientiousness | -.05 | .14 | -.15* | — | -.15** |  |
| Extraversion | -.04 | .02 | -.14** | — | -.14* |  |
| Gender | -.30 | .14 | -.12* | — | -.12 |  |

*Note.* B = unstandardised coefficient. SE = Standard Error. *β =* standardised coefficient.

Indirect and total effects are reported in standardised form. *** *p* < .001, ***p* < .01 **p* < .05.
